# Fungal communities on alpine cheese rinds in Southern Switzerland

**DOI:** 10.1186/s40529-023-00371-2

**Published:** 2023-03-11

**Authors:** Sophie De Respinis, AnnaPaola Caminada, Elisa Pianta, Antoine Buetti-Dinh, Patrizia Riva Scettrini, Liliane Petrini, Mauro Tonolla, Orlando Petrini

**Affiliations:** 1 Institute of Microbiology , University of Applied Sciences and Arts of Southern Switzerland (SUPSI), Via Mirasole 22A, 6500 Bellinzona, Switzerland; 2 Agriculture Advisory Service, Republic and Canton of Ticino, Viale Stefano Franscini 17, 6501 Bellinzona, Switzerland; 3 POLE Pharma Consulting, Via Al Perato 15C, 6932 Breganzona, Switzerland; 4 Breganzona, Switzerland

**Keywords:** High-throughput sequencing, ITS, MALDI-TOF MS, Metabarcoding, MinION, Mycobiome

## Abstract

**Background:**

The biodiversity of the mycobiota of soft cheese rinds such as Brie or Camembert has been extensively studied, but scant information is available on the fungi colonizing the rinds of cheese produced in the Southern Switzerland Alps. This study aimed at exploring the fungal communities present on rinds of cheese matured in five cellars in Southern Switzerland and to evaluate their composition with regards to temperature, relative humidity, type of cheese, as well as microenvironmental and geographic factors. We used macro- and microscopical morphology, matrix-assisted laser desorption/ionization–time of flight (MALDI-TOF) mass spectrometry, and sequencing to characterize the fungal communities of the cheeses, and compared them with metabarcoding targeting the ITS region.

**Results:**

Isolation by serial dilution yielded 201 isolates (39 yeasts and 162 filamentous fungi) belonging to 9 fungal species. *Mucor* and *Penicillium* were dominant, with *Mucor racemosus*, *M. lanceolatus*, *P. biforme*, and *P. chrysogenum/rubens* being the most frequent species. All but two yeast isolates were identified as *Debaryomyces hansenii*. Metabarcoding detected 80 fungal species. Culture work and metabarcoding produced comparable results in terms of similarity of the fungal cheese rind communities in the five cellars.

**Conclusions:**

Our study has shown that the mycobiota on the rinds of the cheeses studied is a comparatively species-poor community influenced by temperature, relative humidity, type of cheese, and manufacturing steps, as well as microenvironmental and possibly geographic factors.

**Supplementary Information:**

The online version contains supplementary material available at 10.1186/s40529-023-00371-2.

## Background

Bloomy, natural or washed cheese rinds host a microbial community composed by bacteria, yeasts, and filamentous fungi (Wolfe et al. 2014; Irlinger et al. 2015; Dugat-Bony et al. 2016; Ceugniez et al. 2017; Fröhlich-Wyder et al. 2019; Choi et al. 2020) that contribute to the development of taste, aroma, texture, and appearance of a given cheese type. The appearance and taste, however, are the result of complex dynamic and biochemical processes that are influenced also by the cheesemaker practices and environmental conditions such as manufacturing temperatures, ripening time, and cellar temperatures (Duru et al. 2018). In addition to environmental factors, however, steps undertaken during the manufacturing process contribute to determine the organoleptic features of cheese. For instance, cheesemakers sometimes add *Penicillium roqueforti* spores to the milk before renneting to produce blue cheeses, or at start of ripening they spray a spore suspension of *P. camemberti* (= *P. candidum*) on the surface, to obtain the bloomy rinds of Camembert or Brie cheeses (Kindstedt 2014). The handling by the cheesemakers during the ripening phase has also been shown to be crucial for the development of the taste and aroma typical for a given cheese type (Banjara et al. 2015; Penland et al. 2021; Sun and D’Amico 2021). For cheese produced from milk of cows and goats grazing on alpine meadows in Southern Switzerland (“Formaggio d’alpe ticinese”, Protected Designation of Origin; henceforth "Ticino alpine cheese"), yeasts and filamentous fungi are never inoculated on the surface; cleaning and turning procedures most likely favor the establishment of specific cheese rind mycobiota originating from the air, the cellar walls and ground, or other hitherto not identified sources, and probably colonizing the rinds during the ripening process. The fungal biodiversity is strongly influenced by the air quality in the cheesemaking rooms, the equipment used, the salting technology, and the micro-ecological conditions in the ripening cellar.

The biodiversity of fungi growing in or on Brie or Camembert cheeses has been extensively investigated (Samson and Eckardt 1977; Nuñez 1978; Marcos et al. 1990; Lund et al. 1995; Ropars et al. 2012; Marcellino and Benson 2013; Wolfe et al. 2014; Banjara et al. 2015; Garnier et al. 2017), but to our knowledge, no studies have been published on the mycobiota colonizing rinds of Ticino alpine cheese manufactured according to the regulations of the Swiss Federal Office for Agriculture (FOAG https://www.blw.admin.ch/blw/it/home/instrumente/kennzeichnung/ursprungsbezeichungen-und-geografische-angaben.html). According to these guidelines (FOAG https://www.blw.admin.ch/blw/it/home/instrumente/kennzeichnung/ursprungsbezeichungen-und-geografische-angaben.html), Ticino alpine cheese must be produced with raw milk, and the finished cheese has to be stored in cellars on spruce or larch boards for at least 60 days before being sold, at a temperature between 10 and 16 °C and a relative humidity (rH) of at least 85%. During storage, the cheese must be cured (turned, cleaned by dry brushing, or rubbing with salt or tap water) on average once a week.

The current climate changes with raised temperatures in the ripening rooms during the summer, structural changes in the buildings that may substantially modify the relative humidity and thus alter the microclimatic conditions of the cellars, as well as the reduction of the amount of salt added to the curd before pressing, may influence the delicate balance between the typical cheese mycobiota and other halotolerant taxa, allowing unwanted taxa to colonise the rinds. In fact, some unexpected filamentous fungi (e.g., *Aspergillus ochraceus*, *Trichothecium roseum*) have been recently isolated from the rinds of Ticino alpine Cheese in some ripening cellars in which the conditions set in the FOAG guidelines were no longer met (L.E. Petrini & O. Petrini, unpublished data).

Ecological investigation of fungal communities is fundamentally changing through the widespread use of omics techniques. Traditionally, the mycobiota of cheese has been studied by fungal isolation in pure culture, and identification using morphological characters and/or molecular markers such as ITS. This method, however, has some drawbacks. For instance, slow-growing fungi are quickly overgrown by fast-growing ones, and not all fungi are culturable, making a reliable determination of species diversity, abundance, and evenness difficult.

The use of high-throughput sequencing (HTS) technologies may allow a more comprehensive characterization of microorganism communities by detecting environmental DNA (eDNA). For instance, metabarcoding of the 16S rDNA of bacteria (Quigley et al. 2012; Wolfe et al. 2014; Dugat-Bony et al. 2016; Duru et al. 2018; Murugesan et al. 2018; Choi et al. 2020; Irlinger and Monnet 2021; Penland et al. 2021) and of the internal transcribed spacer (ITS) region of yeasts and filamentous fungi has been used to characterize the microbial communities of cheeses from different geographical origins (Wolfe et al. 2014; Dugat-Bony et al. 2016; Ceugniez et al. 2017; Murugesan et al. 2018; Irlinger and Monnet 2021; Penland et al. 2021).

This study aims to provide initial information on the mycobiota present on the rinds of Ticino alpine cheeses. We studied the fungal communities of the rinds of cheese produced and ripened as set in the guidelines for “Formaggio d’alpe ticinese” [Swiss Food and Agriculture administration (FOAG)] using serial dilutions and identification of the organisms isolated in pure culture by morphological characters, matrix-assisted laser desorption/ionization–time of flight (MALDI-TOF) mass spectrometry (MS), and DNA sequencing. We also compared the classical isolation methods with metabarcoding targeting the ITS region, to assess whether this technique could be a reliable and rapid tool to be used in ecological studies of fungal communities of cheese rinds.

## Materials and methods

### Sites and sampling

Rind samples were collected from cheeses produced on five different alpine meadows (Table 1), selected according to their geographic position and the type of cheese produced. All cellars had a soil floor and uncured walls, with summer temperatures between 10 and 16 °C and 85% minimum rH; the salt content of the cheese ranged between 5 and 15 g/kg. Cheeses were produced using the same starter culture containing *Lactobacillus casei*, *L. delbrueckii*, *Lactococcus lactis*, and *Streptococcus salivarius*.

**Table 1:** Geographical position of the cellars investigated, and type of cheese produced

Alp, cellar	Code	Location	GPS coordinates	Elevation (m.a.s.l.)*	Milk used	Processing techniques used during ripening
Bolla and Carassina	A	Olivone, Blenio Valley	46°32′36"N 8°58′16"E	1720	Raw cow milk	Turning and rubbing every other day with salt water
Bresciana	B	Olivone, Blenio Valley	46°30′35"N 9°00′22"E	2299	Raw cow milk	Turning each day and dry brushing
Campo la Torba	C	Lavizzara, Maggia Valley	46°29′13"N 8°35′32"E	1859	Raw cow / goat milk	Turning and rubbing with tap water each other day
Formazzora	D	Bedretto, Bedretto Valley	46°28′27"N 8°28′24"E	2141	Raw cow milk	Regular turning and dry brushing
Pontino	E	Airolo, Leventina Valley	46°32′55"N 8°37′23"E	2034	Raw cow milk	Regular turning and dry brushing, rubbing every 3–4 days with salt water

^*^m.a.s.l. meters above sea level

**Fig. 1:**
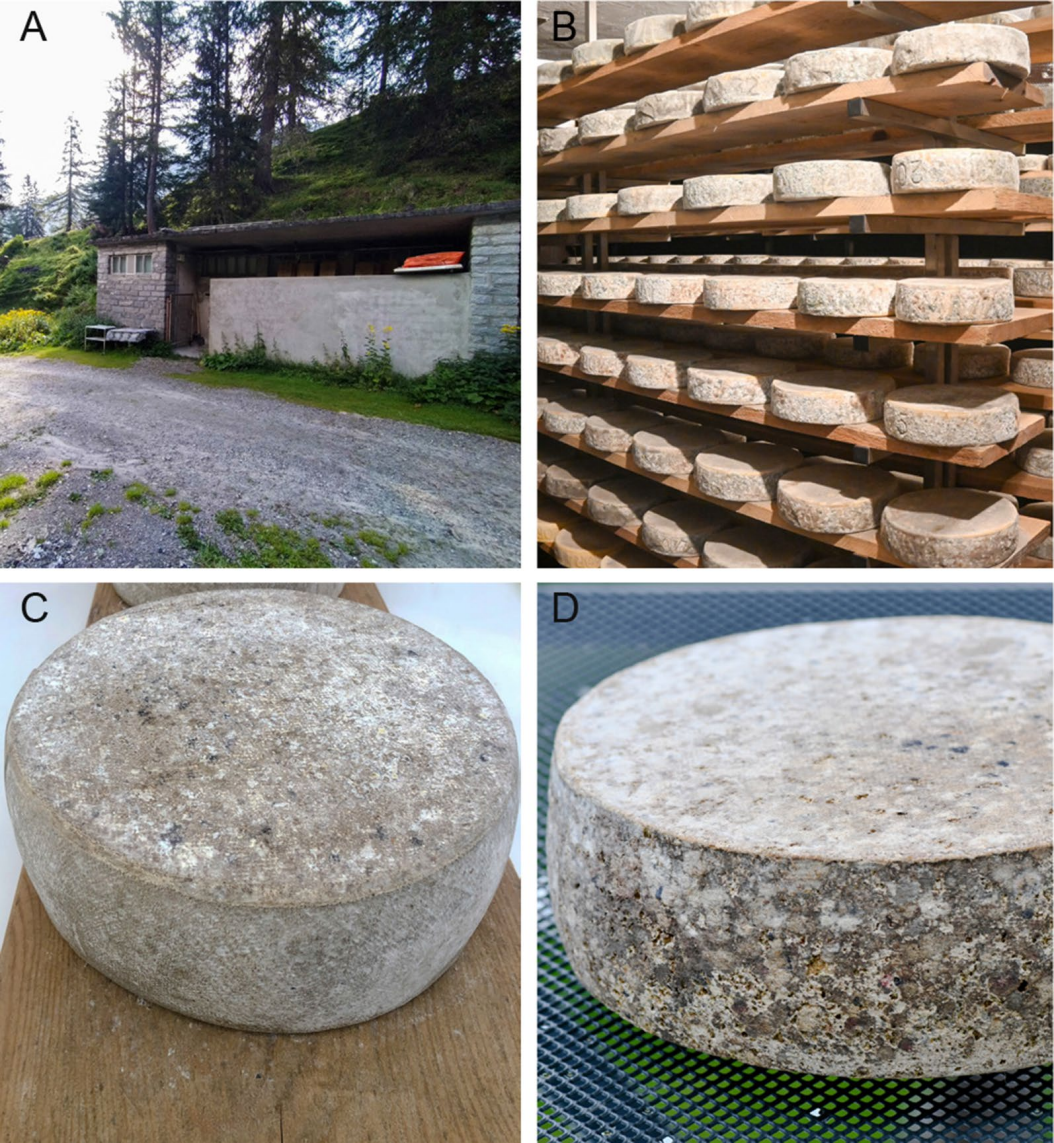
**A** Example of a typical ripening cellar (Cellar D, Formazzora). **B** Interior of Cellar D (Formazzora), showing the cheese storage on larch boards. **C** Close-up of a typical Ticino alpine cheese form. **D** A typical Ticino alpine cheese form at the end of the ripening phase (Cellar C, Campo la Torba)

During the summer 2019, two HOBO Pro v2 data loggers (Onset Computer Corporation, USA) were installed in each cellar to record temperature and relative humidity (rH) to account for any differences observed by the cheesemakers at different locations in the cellars. Pictures of a typical cheese ripening cellar on a Ticino meadow, of the storage methods used, and of the rind surface of a cheese form are presented in Fig. 1.

Samples were taken from a surface of approx. 5 × 5 cm of the cheese rinds by wiping with a sterile swab soaked in 0.8% NaCl. Each swab was placed in a sterile tube for transport and subsequent storage in the laboratory.

### Fungal cultures

One sample from each cellar was used for culture dependent methods and metabarcoding. The swab samples were resuspended in 0.8% NaCl and incubated for 2 h at room temperature under continuous agitation. Aliquots of 10 µl of each suspension were diluted and plated on 2% malt agar (MA; VWR, Belgium) and Dichloran (18%) glycerol agar (DG18; Lab M, United Kingdom) culture media. The plates were checked daily for fungal growth and growing fungal colonies were re-inoculated on Sabouraud Gentamicin Chloramphenicol 2 (SGC2; bioMérieux, France). If more than one species was present on the SGC2 plates, mycelium fragments were re-isolated until a pure colony was obtained. The remaining sample suspension was conserved at −20 °C until metabarcoding analysis.

### Fungal identification by morphology, MALDI-TOF MS, and sequencing

Each colony was inoculated on potato dextrose medium (PDA; Sigma-Aldrich, Switzerland) for morphological identification. SGC2 is not particularly suitable for the development of morphological features of environmental fungi (Scognamiglio et al. 2010) and it was used only for MALDI-TOF MS, as it has proved to be optimal for MALDI-TOF MS analyses of filamentous fungi by reducing their sporulation, thus facilitating protein extraction (De Respinis, unpublished data).

Identification of filamentous fungi was based on macro- and microscopic characters and growth characteristics on PDA. Morphological identification, if needed, was complemented by sequencing, which was carried out only for those filamentous fungi (e.g., *Penicillium* spp.) that could not be confidently identified by their morphological features. DNA was extracted using the phenol–chloroform method (Ausubel et al. 1989) with a 10 mg/ml lysozyme pre-treatment. The ITS region of ribosomal DNA (White et al. 1990) was sequenced for most isolates. We used the *β-tubulin* partial gene (Glass and Donaldson 1995) for a more accurate identification of *Penicillium* species, because the ITS sequences are not diverse enough to satisfactorily delimit species of *Penicillium,* while the *β-tubulin* gene exhibits an appropriate level of divergence among species of this genus (Geiser et al. 2000; Giraud et al. 2010).

All filamentous fungi identified morphologically or by sequencing underwent also MALDI-TOF MS to confirm identification of strains belonging to taxa that were already present in the available database, and to use the generated MALDI-TOF mass spectra to expand the in-house database. At least 3–5 isolates of each species identified by morphological characters and sequencing were utilised for MALDI-TOF MS database construction. The resulting reference spectra (SuperSpectra™) were then used to confirm the identity of all filamentous fungi isolates identified by morphology but not sequenced. MALDI-TOF MS was also the main identification technique used for yeasts because the available spectra databases allow identifying the most common yeasts growing in culture (Patel 2019).

Filamentous fungi were submitted to protein extraction before MALDI-TOF MS as previously described (De Respinis et al. 2014), and yeast isolates underwent a pretreatment with 1 µL 70% formic acid, added to the target plate after direct spotting of the yeast colonies. Samples were spotted in quadruplicate on a MALDI-TOF MS target plate (bioMérieux, France), covered with 1 µl of α-cyano-4-hydroxy-cinnamic acid matrix (CHCA; bioMérieux, France), and air dried. Spectrum acquisition was performed with a VITEK MS RUO mass spectrometer (AXIMA Confidence; bioMérieux, France) equipped with a 50 Hz nitrogen laser (pulse of 3 ns). Mass spectra were collected in positive linear mode in the range of 3000–20,000 mass-to-charge ratio (m/z) with delayed, positive ion extraction (delay time of 104 ns with a scale factor of 800), and an acceleration voltage of 20 kV. For each analysis, 100 averaged profile spectra were collected and processed using the MALDI MS Launchpad 2.9.3 software (bioMérieux, France) with baseline correction, peak filtering, and smoothing procedures.

MALDI-TOF MS identification was performed by comparing the mass spectra obtained with those present in the bioMérieux (France) VITEK^®^ MS SARAMIS^®^ RUO database (Spectral Archiving and Microbial Identification, Version 4.1.0.9), complemented with in-house reference spectra and the MSI database for fungi available online (Normand et al. 2017). Peak lists (m/z 3000 to 20,000) were imported into the VITEK^®^ MS SARAMIS^®^ RUO software and used to produce a dendrogram using a bioMérieux proprietary single linkage agglomerative cluster analysis (0.08% error).

### MinION metabarcoding

The MinION™ sequencer [Oxford Nanopore Technologies (ONT), UK] generates long amplicon reads and it has been already used to study fungal communities (Mafune et al. 2020).

#### DNA extraction and PCR amplification

DNA was extracted from 200 µl of a resuspended sample of each cellar using the FastDNA spin kit for soil (MP Biomedicals, USA), according to the manufacturer’s instructions.

The 2 × KAPA HiFi Hot Start Ready Mix (Roche, Switzerland) was used for PCR, following the manufacturer’s instructions. The amplicons were generated with tailed primers (ONT, UK), to allow further attachment of barcodes. The ITS region was amplified with tailed ITS1 and ITS4 primers (White et al. 1990). The PCR conditions for the ITS region were as follows: first denaturation of 3 min at 95 °C, 35 cycles of denaturation of 20 s at 98 °C, annealing of 15 s at 55 °C, elongation of 30 s at 72 °C, and a last elongation of 1 min at 72 °C.

#### MinION library preparation and multiplexed nanopore sequencing

The amplicons were purified with CleanNGS magnetic beads (Labgene Scientific, Switzerland) and quantified with the Qubit 4 system (Thermo Fisher Scientific, USA). Twenty-four ng (2 pmol) of purified amplicon DNA were used for the barcoding procedure, using barcodes from the PCR Barcoding Expansion 1–96 kit (ONT, UK), and the amplification was carried out with the 2 × KAPA HiFi Hot Start Ready Mix kit (Roche, Switzerland). The cycling conditions were: first denaturation of 3 min at 95 °C, followed by 13 cycles of denaturation of 20 s at 95 °C, annealing of 15 s at 62 °C, elongation of 30 s at 72 °C, and a last elongation of 5 min at 72 °C. The barcoded DNA samples were subjected to a second step of magnetic beads purification and quantification, using the same methods as above, and combined in equal ratios to generate a pool.

One µg of pooled barcoded libraries was finalised using the Ligation Sequencing kit (ONT, UK) and the NEBNext Companion Module for Oxford Nanopore Technologies Ligation Sequencing (New England Biolabs, USA). After a last step of magnetic beads purification and quantification, the libraries were loaded onto a R9.4 flow cell (ONT, UK) using the Flow Cell Priming Kit (ONT, UK).

#### Long-read sequencing

Long-read sequencing was performed with the MinION™ Mk1B sequencer and the Minknow software (21.02.1). Raw fast5 reads were base called using the ONT Guppy Basecalling software (5.0.7) and barcoded using the ONT Guppy Barcoding software (5.0.7), which produced fastq files that were subsequently checked for quality with LongQC (Fukasawa et al. 2020). The taxonomic position of the isolates was then derived analysing the resulting fastq data with the DADA2 (Callahan et al. 2016) R package (1.18), which is considered to yield a comparatively limited number of false detections. The "filterAndTrim" function was run with the arguments minLen = 1200, maxLen = 1600, multithread = TRUE, verbose = TRUE. The "dada" function was run with the parameters OMEGA_A = 1e-10, DETECT_SINGLETONS = TRUE, before taxonomic determination with the "assignTaxonomy" function with the argument tryRC = TRUE.

ITS sequences were then searched against the UNITE database (Nilsson et al. 2019; Kõljalg et al. 2020), sh_general_release_s_10.05.2021.

### Statistical analyses

Species richness, defined as the number of species in a given sample and not as an α-diversity measure as described by Margalef (1958), and evenness were used to describe the ecological parameters of the culture and were mapped graphically to differentiate communities visually (Gauthier and Derome 2021). Whittaker quantile diagrams (Whittaker 1965) displaying relative species richness were also prepared for metabarcoding (read frequencies > 1%) and culture data. Percentages of isolations (for cultural work) or reads of the taxa (for metabarcoding), were also analysed by Multidimensional Scaling (MDS; classical method and L2 dissimilarity) to evaluate similarities among mycobiota from different cellars. A sensitivity analysis was also carried out to evaluate the potential effect of false detections by carrying out MDS of the metabarcoding data on the complete dataset [all operational taxonomic units (OTU) detected] and then on two reduced datasets that included only OTUs detected at frequencies of reads larger than 1% (MDS1) or 2% (MDS2) of the reads. All computations were carried out with Stata version 17 (StataCorp LLC, College Station, Texas, USA), which was used also to prepare graphical displays.

## Results

### Temperature and relative humidity

In the cellars studied, the average temperatures were between 10 and 16 °C at nine out of the ten locations in which dataloggers were placed and were below 10 °C only once at location 2 of Site A (Table 2). The temperatures remained constant over time in all sites and locations, with only few individual values falling below 10 °C and none exceeding 16 °C. Average rH values ranged between 94.5 and 99.8%, and the minimum rH never dropped under 80% (Table 2).

**Table 2:** Temperature (T, °C) and relative humidity (rH, %) measured in the cheese ripening cellars during the study period. Dataloggers were placed in two locations in each cellar (approx. 5 m from each other)

Cellar	Position	T				rH			
		Mean	Median	Min	Max	Mean	Median	Min	Max
A	1	13.59	13.64	12.41	14.72	99.43	100	90.46	100
	2	8.99	8.96	8.22	10.34	99.75	100	83.96	100
B	1	13.21	13.35	11.93	14.98	99.17	100	91.18	100
	2	12.88	13.06	10.71	14.86	99.25	96.46	90.34	100
C	1	12.93	13.30	8.94	15.68	99.74	100	95.27	100
	2	11.75	12.07	7.27	14.46	98.42	100	85.05	100
D	1	13.91	13.95	11.13	15.44	94.53	94.40	85.54	100
	2	13.92	13.69	11.76	16.01	95.45	95.99	85.06	99.01
E	1	13.25	13.26	11.27	14.50	96.13	97.11	80.36	100
	2	13.02	13.16	10.76	13.98	98.53	99.52	87.97	100

### Metabarcoding

Metabarcoding detected 80 fungal species in the samples collected (Table 3 and Additional file 1: Table S1). Fifteen of them were yeasts and the others filamentous fungi (mainly Ascomycota; see Additional file 1: Table S1). Yeasts were predominantly recorded in cellars A and B (geographically close to each other in the Blenio Valley), whereas in cellar C (located in the Maggia Valley and with cheese produced with a mixture of goat and cow raw milk) *Mucoromycota* accounted for 87% of the total number of reads. Cellars D and E, both in the Leventina Valley, had an approximately equal share of yeasts and filamentous fungi.

**Table 3:** Mold and yeast species present at frequencies > 1% of the total number of reads (metabarcoding) or total number of colonies (serial dilutions) in at least one cellar

Number of days between production and sampling	Cellar									
	A		B		C		D		E	
	29		29		23		35		31	
Taxon	M	S	M	S	M	S	M	S	M	S
Yeasts										
*Candida parapsilosis*		2.3								
*Debaryomyces coudertii*^1^	7.27		19.79		0.75		14.58		9.63	
*D. hansenii*		9.3		45.4				26		
*D. prosopidis*^1^	61.80		64.01		1.89		35.83		35.83	
*Trichosporon coremiiforme*				2.4						
Filamentous fungi										
*Mucor lanceolatus*	3.64		2.06	2.4	86.79	70.6	0.41	2	1.60	3.3
*M. plumbeus*			2.31		0.38					
*M. racemosus*				22.7		2.9				
*Penicillium biforme*		74.5		6.8		2.9		42		93.4
*P. chrysogenum/rubens*		11.6		20.4		20.7		22		
*P. concentricum*			0.26				2.08		2.14	
*P. flavigenum*	3.64				0.38		6.25		5.88	
*P. salamii*								2		
*P. thymicola*	14.55		1.28		2.64		20.00		20.85	
*Penicillium* sp.										3.3
Unidentified species		2.3	0.77		0.37	2.9	2.91	6	4.28	

Values are expressed as percentages of the total number of isolates or reads, respectively. M: metabarcoding; S: serial dilution. A: Bolla and Carassina; B: Bresciana; C: Campo la Torba; D: Formazzora; E: Pontino. Database used for the metabarcoding identification: UNITE. ^1^*Debaryomyces coudertii* and *D. prosopidis* have been identified by ITS, but ITS cannot distinguish them from *D. hansenii* (Martorell et al. 2005; Nguyen et al. 2009). *D. hansenii* has been identified by sequencing and MALDI-TOF MS

Except for cellar B, where *Mucor* and *Penicillium* were recorded in similar amounts, each cellar was characterized by the presence of one dominant genus of filamentous fungi. In cellar C, a *Mucor* species identified by ITS metabarcoding as *M.* *lanceolatus* was dominant (Table 3). *Debaryomyces prosopidis* and *D.* *coudertii* were the two most frequent yeasts in cellars A, B, D, and E, whereas only a modest number of reads for these species and for yeasts in general was recorded in cellar C. Based on ITS sequencing, however, *D. coudertii* and *D. prosopidis* cannot be distinguished from *D. hansenii* (Martorell et al. 2005; Nguyen et al. 2009). *Debaryomyces hansenii*, in fact, was identified by MALDI-TOF MS among the isolated yeasts.

**Fig. 2:**
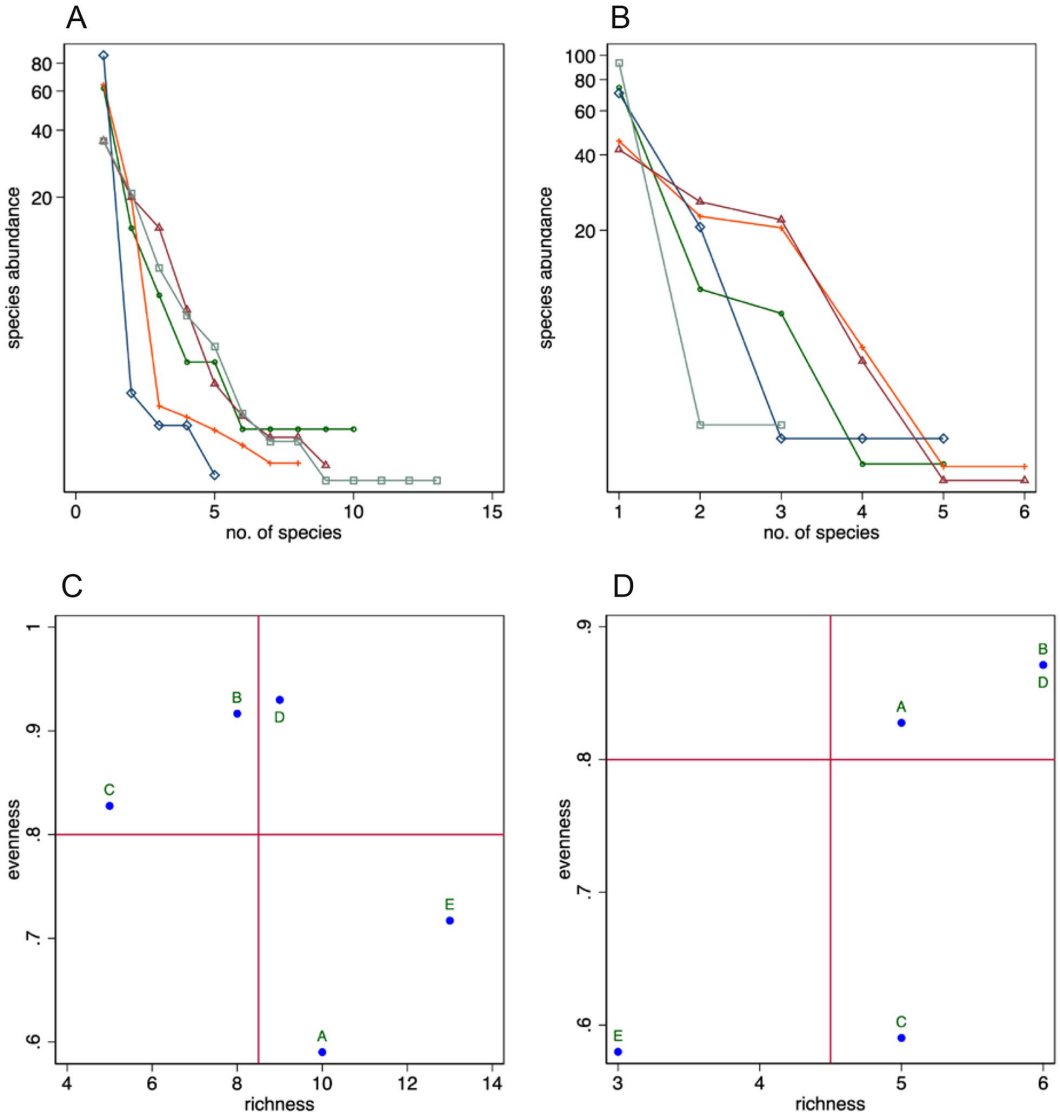
Analysis of the ecological data collected by cultural methods and metabarcoding. In **A** and **B**, green circles indicate cellar A (Bolla and Carassina), orange ticks cellar B (Bresciana), blue diamonds cellar C (Campo La Torba), magenta triangles cellar D (Formazzora), and grey squares cellar E (Pontino). In **C** and **D**, **A**–**E** indicate the cellars as described in Table 1. **A**, **B** Whittaker plots. The number of individuals of each OTU are sorted in descending order; the proportion of the total number of individuals for each species is plotted on the log scale against the OTU rank, presenting the richness of each species ranked from the most (rank 1) to the least frequent rank. The steep plots indicate communities with high dominance. **A** Samples studied by metabarcoding using OTUs with frequencies >1%. **B** Samples studied by cultural methods. **C**, **D** Plot of the evenness vs. richness data for each cellar. **C** Metabarcoding data, using OTUs with frequencies >1%. **D** Culture data

Cellars A and E (Bolla e Carassina and Pontino) account for the highest species richness when metabarcoding is used (Fig. 2A), with 10 and 13 OTUs, respectively, present at frequencies > 1%. In the other three cellars fungal diversity (frequency of reads: > 1%) is lower (8 OTUs in cellar B [Bresciana], 5 in C [Campo La Torba], and 9 in D [Formazzora]), the steep plots indicating communities with high dominance of few species. The loose clusters in Fig. 2C show groupings suggesting an influence of the techniques used during ripening (Table 1) on the fungal community composition.

**Fig. 3:**
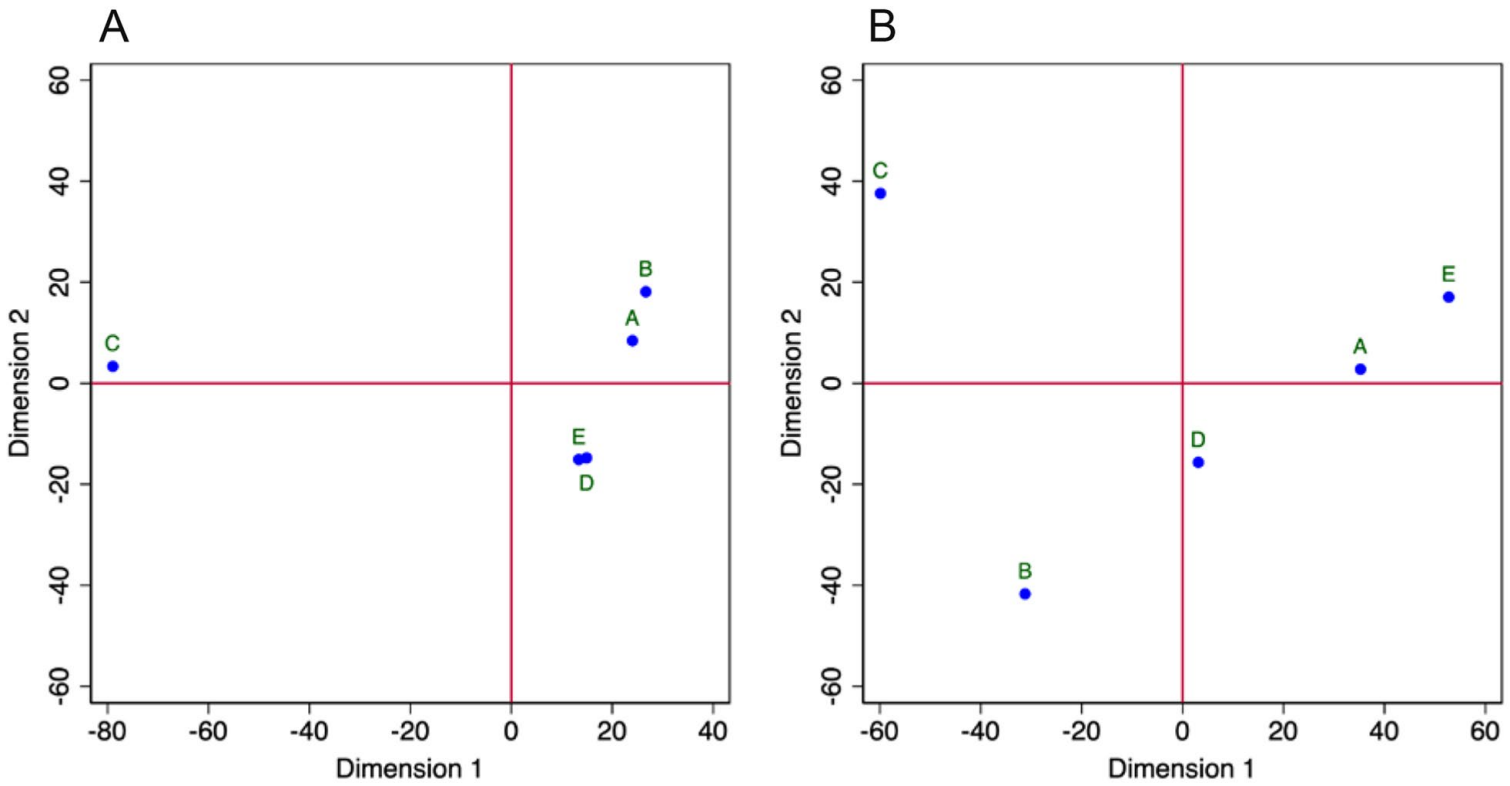
**A** Results of the MDS of the data collected by metabarcoding, computed using only OTUs with frequencies of reads > 1%.** B** Results of the MDS of the data collected by serial dilution. A-E indicate the cellars as described in Table 1. Samples that are geometrically close to each other in the graph are colonised by similar fungal communities

The geographic location of the cellars is consistent with the clustering obtained by MDS (Fig. 3A; Mardia fit measure: 0.99; variance explained by the first 2 dimensions: 98.13%), with cellar C (hosting cheese made from a mixture of goat and cow milk and situated in Maggia Valley) being farther separated from the other cellars located in Blenio (cellars A and B), Bedretto (cellar D), and Leventina (cellar E) valleys. D and E, despite being geographically located in two different valleys, are geographically close (approx. 15 km). The sensitivity analysis (Additional file 1: Fig. S1) provided an almost identical clustering for the MDS analysis with all OTUs (that very likely includes a conspicuous number of false detections) or those present at frequencies > 2%.

### Results from serial dilution isolation

Using the serial dilution method, the cheese rind samples yielded 39 yeasts and 162 filamentous fungi (Table 3 and Additional file 1: Table S2). Morphological characters and ITS, *β-tubulin* gene, and/or translation elongation factor 1α region sequencing allowed distinguishing 9 species. *Mucor* spp. and *Penicillium* spp. were dominant in cellars B and C, whereas in the others only *Penicillium* spp. were frequent (Table 3 and Additional file 1: Table S2). *Mucor racemosus* was most common in cellar B and *M. lanceolatus* in cellar C. *Penicillium biforme* was predominant in cellars A and E, but rare in cellar C, in which *P. chrysogenum/rubens* (species identification uncertain) was more frequent. All but two yeast isolates were identified by MALDI-TOF MS as *Debaryomyces hansenii*.

The Whittaker plot (Fig. 2B) shows a course of the curves with less steep slopes indicating a more uniform species distribution in samples C (evenness: 0.59) and E (0.57). The richness values are low (5 each in cellars A and C, 3 in B, and 6 each in D and E) but these data are hard to be interpreted because of the relatively small sample size. The clustering seen in Fig. 2D confirms the observations made already for the metabarcoding data and suggests that the processing techniques during ripening (Table 1) have a direct influence on the fungal community composition. MDS (Fig. 3B) separates cellar C (Campo la Torba, cheese from mixed cow and goat milk) from all other cellars (Mardia fit measure: 0.9814; percentage explained by the first two dimensions: 98%) but the groupings are not as well defined as with the metabarcoding data. The other cellars (cheese from cow milk only) do not form any compact cluster.

## Discussion

The type of milk utilized for cheesemaking, the techniques used to cure the cheese during ripening, and possibly the geographic location of the cellars, influence the composition of the fungal communities on the rinds of Ticino alpine cheese. This result is only in partial agreement with the outcome of a study of 137 cheese rind samples from 10 different countries, in which similar communities of bacteria and fungi were detected in geographically distinct parts of the world (Wolfe et al. 2014). Wolfe et al. (2014), however, carried out their statistical analysis only at the generic level, whereas we used fungal species as OTUs. ITS metabarcoding generated different OTUs among, but also within genera, and the ecological analysis obtained with theses OTUs provides a more reliable information, the use of genera as OTUs drastically reducing the level of information available to assess between-sample biodiversity.

Ticino, the Southern Switzerland region in which the cellars examined are situated, is small and partitioned lengthwise by four main valleys and high mountain ranges in between, with many alpine meadows where cattle, goats, and sheep graze often together during summertime. We decided to investigate only cellars that complied with the guideline set for the “Protected Designation of Origin” label (FOAG) for Ticino alpine cheese to set a baseline for future studies. Given the apparent microclimatic homogeneity among the different cellars, as suggested by the temperature and relative humidity data collected during the cheese ripening season, we expected similar fungal communities on all Ticino alpine cheese rinds, but data show marked differences at the species and richness level among the five cellars, as highlighted by the MDS analyses (Fig. 3A, B). These differences cannot be conclusively explained: we suspect they may be caused by the type of milk (goat and cow) used for the cheese production and by the different cleaning methods applied during ripening. The geographic location (or perhaps the macro-and microecological conditions at the different locations), however, may also be determinants of the difference.

The species richness of cheese rind microbial communities is lower than that seen in other natural environments, also because of the stress conditions caused by the salty environment (Irlinger et al. 2015). In this study, the cheese rinds with the highest number of species recovered by serial dilutions originated from cellars B and D, and those analysed by metabarcoding from cellar E. Serial dilution yielded at most only 6 species (Fig. 2B), whereas metabarcoding identified 40 (with all OTUs, Additional file 1: Fig. S1). For metabarcoding as well, however, the species richness is reduced to at most 13 (the highest number of species, reported from cellar E; Fig. 2A), if only taxa with more than 1% reads are considered.

*Debaryomyces* was the predominant yeast genus present on all cheese rinds sampled, although it was less frequent in cellar C. It was also often reported from natural and washed rinds in a large study investigating different cheeses from Europe and North America (Wolfe et al. 2014). *D.* *hansenii* is commonly used in the cheesemaking process (Fröhlich-Wyder et al. 2019), but its richness in three of the five cellars studied cannot be explained, because it is never inoculated in the rennet or on the rinds by the local cheesemakers. *D. hansenii*, however, can be present in the raw milk (Quigley et al. 2013) and the favorable environmental conditions during cheesemaking (high salt content, low pH and temperature, lactate as carbon source) may allow colonisation of the cheese surface by this yeast during the early ripening phase (Irlinger et al. 2015; Quijada et al. 2020). *D. hansenii* was dominant in 63% of all cheese types studied by Banjara et al. (2015), and, together with *Geotrichum candidum*, it was detected in 12 French cheese varieties (Dugat-Bony et al. 2016). Quijada et al. (2020) also reported *Debaryomyces* from all rind samples they studied.

The identification of *Debaryomyces* species deserves a brief comment. MALDI-TOF MS identified all isolates as *D. hansenii*, whereas ITS metabarcoding indicated in the samples the presence of *D. prosopidis* and *D. coudertii*, but not *D. hansenii*. Contrarily to the MALDI-TOF MS database used in this study, however, ITS sequencing does not distinguish *D. coudertii* from *D. hansenii* or *D. prosopidis* (Martorell et al. 2005; Nguyen et al. 2009). We thus expect all *Debaryomyces* isolates to belong to *D. hansenii*.

As already reported in other studies on cheese mycobiota, *Mucor* spp. and *Penicillium* spp. were the most common filamentous fungi on the cheese rind samples examined, *Mucor* being the second most prominent genus after *Penicillium*. *Mucor* *lanceolatus* and *M. racemosus*, detected by serial dilution techniques and metabarcoding, were also among six *Mucor* species isolated from French cheeses (Hermet et al. 2012) and were reported from Saint-Nectaire cheese by Dugat-Bony et al. (2016). A species of *Mucor*, very likely *M. racemosus*, is considered a contaminant of alpine cheeses, with a negative impact on the taste and becoming problematic when its occurrence is chronic and persistent (Pillonel 2006). *Mucor* species were not mentioned in the Wolfe et al. (2014) study that analyzed bloomy, natural or washed rinds of European and North American cheeses. One can speculate that not enough humidity was present in the samples studied, because Wolfe et al. (2014) reported some *Scopulariopsis* species that are considered xerophilic (Domsch et al. 2007). Selected *Mucor* strains are used to ripen some semi-soft cheeses including Saint Nectaire, Tomme de Savoie, or Taleggio (Fox and McSweeney 2004) by producing lipases and proteases that contribute to the distinctive flavors, texture, or nutritional quality of these cheeses (Hermet et al. 2012). *Mucor fuscus* is very common (Petrini L.E., unpublished data) on some types of semi-soft cheeses produced in Southern Switzerland and stored in cellars that are very similar to those we studied, yet this species was not recorded from our samples, probably because their water content was not suitable for this taxon to compete with other faster growing *Mucor* species.

*Penicillium* species may enter cheese production facilities or ripening rooms as contaminants and colonise the cheese surfaces (Nielsen et al. 1998; Ropars et al. 2012; Bodinaku et al. 2019). *Penicillium* cultures are sometimes added to the cheese curd during the cheese making process, but none was added to any of the cheeses studied, thus they most probably originated from the manufacturing or ripening environments. *Penicilllium biforme* was frequently isolated by serial dilutions (Table 3 and Additional file 1: Table S2) and it was comparatively rare only in samples from cellar B. MALDI-TOF MS classified this taxon as *P. camemberti*, but β-tubulin sequencing identified it as *P. biforme*, a species considered to be distinct from *P. camemberti* (Giraud et al. 2010) but not distinguishable from *P. fuscoglaucum* with the methods used here. In a whole genome-based analysis *P. biforme*, *P. camemberti*, and *P. fuscoglaucum* formed separate and specific genetic clusters, with *P. biforme* and *P. camemberti*, however, being sister clades (Ropars et al. 2020). Thus, these three species represent closely related but different lineages that have evolved traits beneficial for cheese-making.

Some fungal species such as *Scopulariopsis flava, S. fusca* or *Mammaria* sp. have often been observed on Ticino alpine cheese (L.E. Petrini, unpublished data) but were not recorded during this study. Probably they emerge later in the ripening process when the cheese surface offers a drier environment, hampering fast growing *Mucor* and *Penicillium* species.

In this study we used a combination of culture-dependent and independent methods (Fröhlich-Wyder et al. 2019) because both systems are considered complementary and not contradictory or exclusive (Irlinger et al. 2015). Both produced similar results in terms of dominant taxa present in the samples and similarity of the fungal communities inhabiting the cheese rinds (Table 3, Additional file 1: Table S1). Culture-based methods are difficult to carry out in large-scale studies; they are more demanding and often require selective media. In addition, they are more expensive and time consuming compared to metabarcoding. Non-culturable species cannot be detected, and slow growing strains are easily overgrown by fast-growing ones (Banjara et al. 2015). Metabarcoding, on the other hand, is very sensitive and cost-effective, but it does not distinguish between colonizing species and occasional, dormant contaminants. In addition, metabarcoding is known to yield false negative and false positive detections of species in environmental samples (Buxton et al. 2021). Several techniques have been described to obviate, at least partly, this drawback (e.g., Ficetola et al. 2016; Lahoz-Monfort et al. 2016; Buxton et al. 2021; Polanco Fernández et al. 2021)), and some analysis tools are relatively robust with regards to false detections. In previous studies, for instance, DADA2 (used in this study) has identified more real variants and output fewer spurious sequences than other methods in studies of mock communities (Callahan et al. 2016). An overall knowledge of the mycobiota present on the rind of cheese ripening under controlled standard conditions (“the ground truth”) is needed to reduce the number of false detections, although our sensitivity analysis suggests that a selection of reads present at frequencies higher that a given cutoff (in our case 1%) may contribute to solving the false detection problem.

Our study has some limitations. Because of economic reasons and lacking manpower, the number of cellars and rind samples studied was small. The cheese rinds were sampled only once at the early-mid ripening phase (on average 30 days after production, Table 3) and metabarcoding was carried out on only one sample, precluding the possibility to detect intra-cellar variation of the mycobiota. The use of ITS metabarcoding for the mycobiome is also a limitation, as ITS does not reliably identify several fungal species and the available databases are far from being complete. On the other hand, the exact taxonomic position of the OTUs detected in an ecological study is not crucial to investigate differences in the community composition of different samples, and the sensitivity analysis carried out in our study has shown that even without the elimination of potential false detections (Additional file 1: Fig. S1) the results obtained by metabarcoding are comparable to those derived from cultural methods.

It is known that the dynamic of bacterial and fungal microbiota evolves throughout the ripening process (Quijada et al. 2020; Penland et al. 2021): future studies should foresee sampling during the early and late ripening stages to gather information on the evolution of the rind communities throughout the cheese maturation period.

## Conclusions

The data gathered have shown that the mycobiota community on the rinds of Ticino alpine cheese depends on different parameters, including not only temperature, relative humidity, and type of milk but also manufacturing steps, microenvironmental, and possibly geographic factors.

## Supplementary Information

**Additional file 1: Table S1.** Frequency (in percentage of total number of reads) of mold and yeast species identified by ITS metabarcoding. Due to the inadequacy of ITS to identify some fungal species, species names (in particular *Penicillium* spp.) are not reliable and should be seen only as indicative of the biodiversity observed by metabarcoding. Some species reported may also be false positive detections. **Table S2.** Frequency of mold and yeast species [n, (%)] isolated in pure culture. *Penicillium* spp. were identified using the β-tubulin gene region (Houbraken et al 2020). For *P. biforme* see discussion in the main text. **Fig. S1.** Analysis of the ecological data collected by metabarcoding. **A**: Whittaker plots of all OTUs, presenting the abundance of each OTU ranked from the most (rank 1) to the least frequent rank, for all cellars studied. **B**: Whittaker plots of the OTUs with frequency of reads>2%, presenting the abundance of each species ranked from the most (rank 1) to the least frequent. **C**: Results of the MDS of the data collected by metabarcoding, all OTUs included in the analysis.** D**: Results of the MDS of the data collected by metabarcoding, with only OTUs with frequencies >2% included in the analysis. For additional details see text.

## Data Availability

The datasets generated during and/or analyzed during the current study are available from the corresponding author upon reasonable request.
