# Supplementary material for: Fungal communities on alpine cheese rinds in Southern Switzerland
Source: Bot Stud. 2023 Mar 11;64:6. doi: 10.1186/s40529-023-00371-2 (PMC10008522; doi:10.1186/s40529-023-00371-2)
Supplement: Supplementary file 1 — Additional file 1: Table S1. Frequency (in percentage of total number of reads) of mold and yeast species identified by ITS metabarcoding. Due to the inadequacy of ITS to identify some fungal species, species names (in particular Penicillium spp.) are not reliable and should be seen only as indicative of the biodiversity observed by metabarcoding. Some species reported may also be false positive detections. Table S2. Frequency of mold and yeast species [n, (%)] isolated in pure culture. Penicillium spp. were identified using the β-tubulin gene region (Houbraken et al 2020). For P. biforme see discussion in the main text. Fig. S1. Analysis of the ecological data collected by metabarcoding. A: Whittaker plots of all OTUs, presenting the abundance of each OTU ranked from the most (rank 1) to the least frequent rank, for all cellars studied. B: Whittaker plots of the OTUs with frequency of reads>2%, presenting the abundance of each species ranked from the most (rank 1) to the least frequent. C: Results of the MDS of the data collected by metabarcoding, all OTUs included in the analysis. D: Results of the MDS of the data collected by metabarcoding, with only OTUs with frequencies >2% included in the analysis. For additional details see text. [file 40529_2023_371_MOESM1_ESM.docx]

# Supplementary Material

Table S1. Frequency (in percentage of total number of reads) of mold and yeast species identified by ITS metabarcoding. Due to the inadequacy of ITS to identify some fungal species, species names (in particular *Penicillium* spp.) are not reliable and should be seen only as indicative of the biodiversity observed by metabarcoding. Some species reported may also be false positive detections.

|  | **Cellar** | | | | |
| --- | --- | --- | --- | --- | --- |
| **Taxon** | **A** | **B** | **C** | **D** | **E** |
| **Yeasts and other fungi** | | | | | |
| *Candida aurita* |  |  |  | 0.42 |  |
| *C. hyderabadensis* | **1.82** |  |  |  |  |
| *C. zeylanoides* |  | 0.51 |  |  |  |
| *Cutaneotrichosporon curvatum* |  | 0.77 |  |  |  |
| *Debaryomyces coudertii*^1^ | **7.27** | **19.79** | 0.75 | **14.58** | **9.63** |
| *D. macquariensis* |  |  |  |  | 0.54 |
| *D. prosopidis*^1^ | **61.8** | **64.01** | **1.89** | **35.83** | **35.83** |
| *Debaryomyces* sp.^2^ |  | 0.51 |  | 0.42 |  |
| *Filobasidium stepposum* |  |  |  |  | 0.53 |
| *Hanseniaspora clermontiae* |  | 0.26 |  |  |  |
| *Itersonilia pannonica* |  | 0.26 |  | 0.42 |  |
| *Meyerozyma smithsonii (=C. smithsonii)* |  | **1.54** |  |  | 0.53 |
| *Rhodotorula dairenensis* |  | 0.77 |  | 0.42 |  |
| *Trichosporon insectorum* |  | **1.8** |  |  |  |
| *Ustilago panici-gracilis* |  |  | 0.38 |  | 0.54 |
|  |  |  |  |  |  |
| **Filamentous mucoromycota, ascomycetous and basidiomycetous fungi** | | | | | |
| *Alternaria aspera* |  |  |  | 0.42 |  |
| *A. betae-kenyensis* |  | 0.26 | 0.38 | 0.41 | **1.07** |
| *Ascochyta herbicola* |  |  |  |  | 0.53 |
| *Aspergillus cibarius* |  |  |  | 0.42 |  |
| *As. conicus* |  |  |  | 0.41 |  |
| *As. glabripes* |  |  |  | 0.42 |  |
| *Aureobasidium namibiae* |  |  | 0.37 |  |  |
| *Bisifusarium domesticum* |  |  | **1.89** |  |  |
| *Botryotinia pelargonii* |  |  |  |  | 0.54 |
| *Candolleomyces trinitatensis* (= *Psathyrella trinitatensis*) |  |  |  | 0.42 |  |
| *Cladosporium cycadicola* |  | 0.26 |  | 0.42 | 0.54 |
| *C. exasperatum* |  |  |  | 0.41 |  |
| *C. langeronii* |  |  |  |  | **1.07** |
| *Crustodontia chrysocreas* |  |  |  |  | 0.54 |
| *Curvularia buchloes* |  | 0.26 |  |  |  |
| *Epicoccum dendrobii* |  |  | 0.38 | 0.42 | 0.54 |
| *Erysiphe euonymicola* |  |  |  | 0.41 |  |
| *Fomes fomentarius* (*= F. inzengae*) |  |  |  |  | 0.54 |
| *Funalia floccosa* |  |  |  | 0.83 |  |
| *Gloeocystidiellum kenyense* |  |  |  | 0.42 |  |
| *Hyphodermella rosae* |  |  | 0.75 |  |  |
| *Laetisaria roseipellis* |  |  |  | 0.42 |  |
| *Melampsora iranica* |  |  |  |  | 0.54 |
| *Microdochium novae-zelandiae* |  |  |  | 0.42 |  |
| *Mucor lanceolatus* | **3.64** | **2.06** | **86.79** | 0.41 | **1.60** |
| *M. plumbeus* |  | **2.31** | 0.38 |  |  |
| *Mycena alphitophora* |  |  |  | 0.42 |  |
| *Mycosphaerella tassiana* |  |  |  |  | **1.60** |
| *Penicillium bialowiezense* |  | **1.28** |  |  |  |
| *P. compactum* |  |  |  | 0.42 |  |
| *P. concentricum* |  | 0.26 |  | **2.08** | **2.14** |
| *P. flavigenum* | **3.64** |  | 0.38 | **6.25** | **5.88** |
| *P. gladioli* |  |  |  |  | **1.07** |
| *P. halotolerans* |  |  | 0.38 |  |  |
| *P. infrapurpureum* |  |  | **1.13** | **1.67** | 0.54 |
| *P. kewense* |  |  |  |  | 0.53 |
| *P. kongii* |  |  |  | 0.41 |  |
| *P. roseopurpureum* |  |  |  | 0.42 |  |
| *P. samsonianum* |  |  |  | 0.42 | **1.07** |
| *P. thymicola* | **14.55** | **1.28** | **2.64** | **20.00** | **20.85** |
| *P. tricolor* | **1.82** |  |  | 0.42 |  |
| *P. virgatum* |  |  |  |  | 0.53 |
| *Peniophorella pubera* | **1.82** |  |  |  |  |
| *Perenniporia vanhulleae* |  |  |  |  | 0.53 |
| *Phanerochaete livescens* |  |  |  |  | 0.53 |
| *Phlebia rufa* |  | 0.26 | 0.38 | 0.42 |  |
| *Ph. tremellosa* |  |  |  |  | 0.53 |
| *Pseudopithomyces rosae* |  | 0.26 |  | 0.41 |  |
| *Puccinia aizazii* |  |  |  | 0.41 |  |
| *Pycnoporus cinnabarinus* (= *Trametes cinnabarina*) |  |  |  |  | 0.54 |
| *Radulomyces molaris* |  | 0.26 |  |  | 0.53 |
| *Sarcomyxa serotina* (= *Panellus serotinus*) |  |  |  | 0.42 |  |
| *Sistotremastrum guttuliferum* |  |  |  | 0.42 |  |
| *Stachybotrys chartarum* |  |  |  |  | 0.54 |
| *Symmetrospora symmetrica* |  |  |  |  | 0.53 |
| *Toxicocladosporium irritans* | **1.82** |  |  |  |  |
| *Trametes trogii* |  |  |  | **1.25** | **1.07** |
| *T. villosa* |  |  |  |  | 0.53 |
| *Trechispora hymenocystis* |  |  |  |  | 0.53 |
| *Vuilleminia pseudocystidiata* |  | 0.26 |  |  |  |
| *Wallemia canadensis* |  |  |  | **1.67** |  |
| *W. muriae* |  |  | 0.38 |  |  |
| *Xylodon sambuci* | **1.82** |  |  | 0.83 |  |
| *Yuchengia narymica* |  |  | 0.38 |  | 0.54 |
|  |  |  |  |  |  |
| unidentified species |  | 0.77 | 0.37 | **2.91** | **4.28** |
|  |  |  |  |  |  |

A: Bolla and Carassina; B: Bresciana; C: Campo la Torba; D: Formazzora; E: Pontino. Database used for the metabarcoding identification: UNITE. In bold, species present at frequencies >1%. ^1^*Debaryomyces coudertii* and *D. prosopidis* have been identified by ITS, but ITS cannot distinguish these two taxa from *D. hansenii* (Martorell et al. 2005; Nguyen et al. 2009).

Table S2. Frequency of mold and yeast species [n, (%)] isolated in pure culture. *Penicillium* spp. were identified using the β-tubulin gene region (Houbraken et al 2020). For *P. biforme* see discussion in the main text.

|  | **Cellar** | | | | |
| --- | --- | --- | --- | --- | --- |
| **Taxon** | **A** | **B** | **C** | **D** | **E** |
| **Yeasts** |  |  |  |  |  |
| *Candida parapsilosis* | 1 (2.3) |  |  |  |  |
| *Debaryomyces hansenii*^1^ | 4 (9.3) | 20 (45.4) |  | 13 (26) |  |
| *Trichosporon coremiiforme* |  | 1 (2.4) |  |  |  |
|  |  |  |  |  |  |
|  |  |  |  |  |  |
| **Filamentous fungi** |  |  |  |  |  |
| *Penicillium biforme* | 32 (74.5) | 3 (6.8) | 1 (2.9) | 21 (42) | 28 (93.4) |
| *P. chrysogenum/rubens* | 5 (11.6) | 9 (20.4) | 7 (20.7) | 11 (22) |  |
| *P. salamii* |  |  |  | 1 (2) |  |
| *Penicillium* sp. (Sect. *Chrysogena*) |  |  |  |  | 1 (3.3) |
| *Mucor lanceolatus* |  | 1 (2.4) | 24 (70.6) | 1 (2) | 1 (3.3) |
| *M. racemosus* |  | 10 (22.7) | 1 (2.9) |  |  |
|  |  |  |  |  |  |
| Unidentified filamentous species | 1 (2.3) |  | 1 (2.9) | 3 (6) |  |
| Total | 43 | 44 | 34 | 50 | 30 |

A: Bolla and Carassina; B: Bresciana; C: Campo la Torba; D: Formazzora; E: Pontino. ^1^*D. hansenii* has been identified by sequencing and MALDI-TOF MS.

Fig. S1. Analysis of the ecological data collected by metabarcoding. **A**: Whittaker plots of all OTUs, presenting the abundance of each OTU ranked from the most (rank 1) to the least frequent rank, for all cellars studied. **B**: Whittaker plots of the OTUs with frequency of reads>2%, presenting the abundance of each species ranked from the most (rank 1) to the least frequent. **C**: Results of the MDS of the data collected by metabarcoding, all OTUs included in the analysis. **D**: Results of the MDS of the data collected by metabarcoding, with only OTUs with frequencies >2% included in the analysis. For additional details see text.
